# Relationship between Resilience, Psychological Distress and Physical Activity in Cancer Patients: A Cross-Sectional Observation Study

**DOI:** 10.1371/journal.pone.0154496

**Published:** 2016-04-28

**Authors:** Martin Matzka, Hanna Mayer, Sabine Köck-Hódi, Christina Moses-Passini, Catherine Dubey, Patrick Jahn, Sonja Schneeweiss, Manuela Eicher

**Affiliations:** 1 Department of Nursing Science, University of Vienna, Vienna, Austria; 2 School of Health Fribourg, University of Applied Sciences and Arts Western Switzerland, Fribourg, Switzerland; 3 Institute for Health and Nursing Science, Nursing Research Unit, University Hospital Halle (Saale), Halle (Saale), Germany; 4 Directorate of Nursing, Department of Organizational Development, Vienna General Hospital, Vienna, Austria; 5 Institute of Higher Education and Research in Nursing. Faculty of Biology and Medicine, University of Lausanne, Lausanne, Switzerland; The George Institute for Global Health, INDIA

## Abstract

**Objective:**

Psychological distress remains a major challenge in cancer care. The complexity of psychological symptoms in cancer patients requires multifaceted symptom management tailored to individual patient characteristics and active patient involvement. We assessed the relationship between resilience, psychological distress and physical activity in cancer patients to elucidate potential moderators of the identified relationships.

**Method:**

A cross-sectional observational study to assess the prevalence of symptoms and supportive care needs of oncology patients undergoing chemotherapy, radiotherapy or chemo-radiation therapy in a tertiary oncology service. Resilience was assessed using the 10-item Connor-Davidson Resilience Scale (CD-RISC 10), social support was evaluated using the 12-item Multidimensional Scale of Perceived Social Support (MSPSS) and both psychological distress and activity level were measured using corresponding subscales of the Rotterdam Symptom Checklist (RSCL). Socio-demographic and medical data were extracted from patient medical records. Correlation analyses were performed and structural equation modeling was employed to assess the associations between resilience, psychological distress and activity level as well as selected socio-demographic variables.

**Results:**

Data from 343 patients were included in the analysis. Our revised model demonstrated an acceptable fit to the data (χ^2^(163) = 313.76, *p* = .000, comparative fit index (CFI) = .942, Tucker-Lewis index (TLI) = .923, root mean square error of approximation (RMSEA) = .053, 90% CI [.044.062]). Resilience was negatively associated with psychological distress (*β* = -.59), and positively associated with activity level (*β* = .20). The relationship between resilience and psychological distress was moderated by age (*β* = -0.33) but not social support (*β* = .10, *p* = .12).

**Conclusion:**

Cancer patients with higher resilience, particularly older patients, experience lower psychological distress. Patients with higher resilience are physically more active. Evaluating levels of resilience in cancer patients then tailoring targeted interventions to facilitate resilience may help improve the effectiveness of psychological symptom management interventions.

## Background

Cancer patients often suffer simultaneously from multiple symptoms related to their disease or treatment including fatigue, disturbed sleep, pain, nausea, lack of appetite and neuropathy. These symptoms and resulting functional impairment can cause distress, reduce health-related quality of life (HRQOL) [1] and may limit treatment options [2]. Further, increases in the number and/or intensity of symptoms are associated with reduced overall survival time [3]. Clinically, the cumulative severity and impact of symptoms reported by a significant proportion of patients with a given tumor entity or treatment has been defined as «symptom burden » [4]. Notably, symptom appraisal is influenced by a variety of factors including demographic/sociocultural characteristics, developmental stage, psychological/physiological characteristics, as well as individual health and illness factors [5]. This might help explain why cancer patients with similar diagnoses and treatment status have significantly different levels of symptom distress, a fact that may also be attributed to the concept of resilience [6].

Resilience influences symptom appraisal and the experience of patients with cancer [7, 8]. Resilience has been defined as resistance, recovery, or rebound of mental and physical health after a challenge [9]. For adult cancer patients, resilience is described as a dynamic process of facing adversity related to a cancer experience that can be facilitated through interventions [6]. Besides biological factors (e.g. gene-environment interactions) and personal factors (e.g. self-efficacy, flexibility, optimism), environmental factors -most notably social support -contribute to an individual’s resilience and consequently to favorable mental and physical patient outcomes [10]. However, the most commonly employed and most widely translated and validated measurement scales for resilience tend to focus primarily on personal factors [6].

To date, there are limited data on the relationship between resilience and psychological distress in cancer patients during treatment. High resilience scores have been shown to be associated with less anxiety and depression in samples of cancer survivors [7, 11, 12], as well as in cancer patients undergoing treatment [13]. Conversely, lower levels of resilience predict impaired psychological functioning, and also predict fatigue among patients with cancer [8, 14]. Yet, only two of the studies cited took social support into account [7, 13]), of which only one was conducted in cancer patients undergoing treatment, showing a negative association of social support and psychological distress [13]. Thus, little is known about the association and potential interaction of resilience and social support in relation to mental health in this particular patient group. Evidence on resilience and activity levels equally remains sparse. A few studies on aging adults and patients with Parkinson disease point to a protective role for resilience in relation to disability and the ongoing ability to complete activities of daily living [15, 16]. In survivors of stem cell transplantation (mostly cancer survivors), patients with higher resilience scores reported significantly better physical functioning than those scoring lower on resilience [12] and resilience was associated with less severe graft-versus-host disease, as well as less permanent disability [17]. Resilience has also been linked to better physical functioning in long-term cancer survivors [18]. However, to our knowledge, the relationship between resilience and impairment of physical activity in cancer patients undergoing treatment has yet to be investigated.

While older age is associated with increased limitations in activities of daily living [15], it is also frequently linked to lower emotional distress (i.e. anxiety and depression) in patients with cancer [19]. Current evidence on the association between age and resilience is conflicting and inconclusive. Some studies have shown resilience to be either increased or decreased with older age [19, 20] while others have demonstrated that there is no clear relationship [21]. As noted previously, social support contributes to resilience in general, but may be a particularly important factor contributing to resilience in older age while facing physical and mental adversity [22]. Thus, age and social support may be relevant covariates of psychological distress and activity levels, while their interaction with resilience remains unclear.

**Fig 1:**
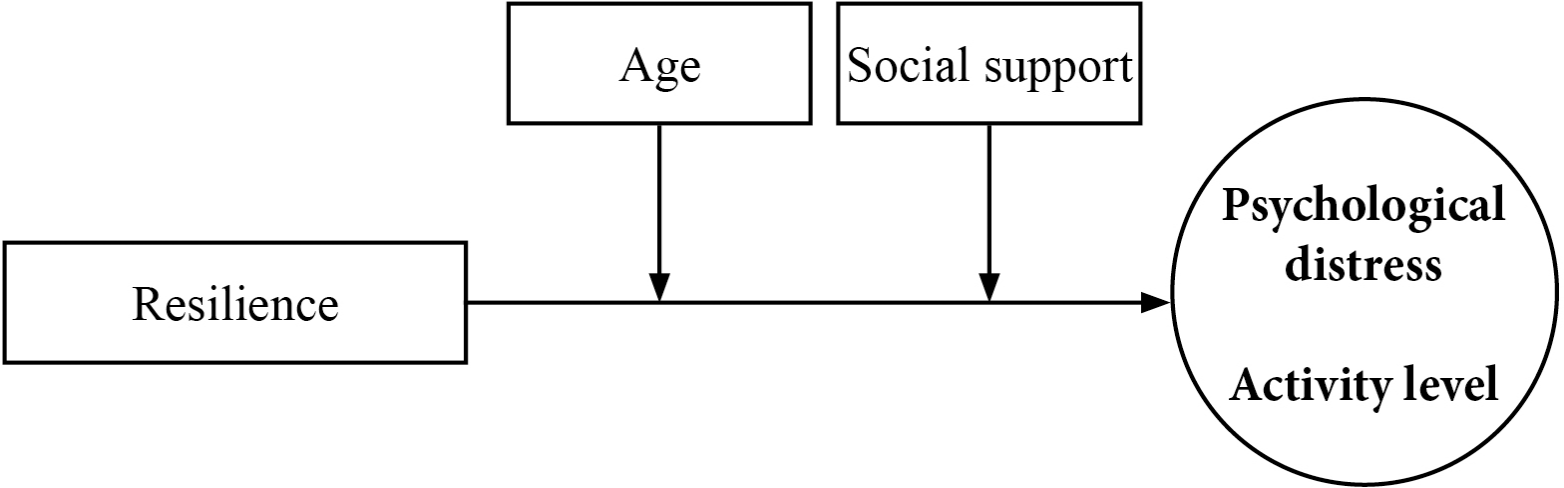
Conceptual diagram. Proposed relationship between resilience (Connor-Davidson Resilience Scale), psychological distress and activity level (Rotterdam Symptom Checklist), with age and social support as moderators.

We hypothesized that there is an association of resilience and both psychological distress and activity level, which is moderated by age and social support (Fig 1). Therefore, to better understand the relationship between resilience, psychological distress, and physical activity in adult patients undergoing cancer treatment, we tested a structural equation model of these variables.

## Method

This study was part of a larger cross-sectional observation study conducted for quality assurance in several ambulatory and inpatient oncology departments of a tertiary university medical center (Vienna, Austria). The ethics commission of the Medical University of Vienna reviewed and approved the study protocol (Nr. 1223/2014). After being informed in detail about the study aims and procedures, patients provided oral informed consent prior to study participation.

Data are reported according to the STROBE guidelines (Strengthening the Reporting of Observational Studies in Epidemiology) for reporting observational studies [23].

### Sample and Setting

Study subjects included a convenience sample of adult (≥ 18 years-old) patients diagnosed with cancer who were undergoing chemotherapy, radiotherapy or chemo-radiation at the Vienna General Hospital (Austria). Participants were judged by their clinicians to be mentally and physically capable of participating in the study. Study recruitment was conducted over a 2-week period in May 2014 by nursing staff who had completed a 5-hour training course on the study protocol and study-related activities. Initial power estimates indicated a target of 400 participants was needed to ensure adequate power for the proposed analyses.

### Measurements

Socio-demographic data were collected and disease-specific data regarding tumor site, treatment modality, treatment cycle and stage of disease (locally advanced, metastatic or recurrent cancer) were gathered from the medical records.

Resilience was assessed using the author-approved German translation of the 10-item Connor-Davidson Resilience Scale (CD-RISC 10). This instrument provides a unidimensional measure reflecting the ability to bounce back from a variety of challenges such as illness, emotional pressure or painful feelings. Items are rated on a 5-point scale (0 = “not true at all” to 4 = “true nearly all the time”) providing a total sum score ranging from 0–40, with higher scores reflecting greater resilience [24]. The German translation of the CD-RISC 10 has acceptable psychometric properties with high internal consistency (Cronbach’s alpha = .84) [25]. Social support was measured using the 12-item Multidimensional Scale of Perceived Social Support (MSPSS). This instrument assesses the perceived adequacy of social support from family, friends and significant others. To ensure consistency within the survey, we transformed the original 7-point scale into a 5-point scale. Accordingly, cumulative scores range from 12–60, with higher scores reflecting greater perceived social support. Internal consistency of the original scale is high (Cronbach’s alpha = 0.88) and construct validity is adequate [26]. The MSPSS was translated into German (for- and backward) and culturally adapted following the guidelines of the ISPOR (International Society for Pharmacoeconomics and Outcomes Research) [27]. Compared to the original scale, the internal consistency of this German translation of the MSPSS is slightly higher in our sample (Cronbach’s alpha = 0.92). Psychological distress and activity level were assessed using the author-approved German translation of the Rotterdam Symptom Checklist (RSCL). Briefly, the RSCL is a 39-item self-report questionnaire designed specifically for patients with cancer. It measures quality of life across four domains: physical symptom distress, psychological distress, activity level, and overall global quality of life [28]. Patients rate the extent to which they have been bothered by each of the 30 symptoms during the past week (not at all, a little, quite a bit, or very much). The German translation of the RSCL has acceptable psychometric properties and high internal consistency for the physical and psychological distress subscales (Cronbach’s alphas = 0.85), as well as the activity level subscale (Cronbach’s alphas = 0.89) [29]. For the present study we utilized two subscales, psychological distress (7-items, Cronbach’s alpha = 0.86) and activity level (8-items, Cronbach’s alpha = 0.91). Higher scores indicate greater psychological distress and lower levels of impaired physical activity respectively.

### Statistical Analysis

Patient characteristics were summarized using descriptive statistics. Correlation analysis was conducted for psychological distress, activity level, resilience and social support scales, as well as medical and sociodemographic data. Statistically significant covariate variables were included in modeling to test the impact of resilience, age, social support and the interaction of resilience with both age and social support on psychological distress and activity impairment respectively. Resilience, age and social support variables were mean centered. After testing of the measurement model, we tested two structural equation models (SEM) using an exploratory structural factor model (ESEM) approach that integrates exploratory factor analysis within a structural equation framework. That is, the measurement model was incorporated in the two SEM models. The initial SEM model tested included three covariates (income, gender, and work status), in addition to our main predictors: social support, age, resilience and their moderator variables. We derived a revised model by excluding the non-significant covariates in the initial model. The ESEM approach is more flexible for testing *a priori* hypotheses regarding an expected factor structure. In contrast to confirmatory factor analysis, ESEM cross-loading between items is not assumed to be zero. We thus examined the two-factor solution underlying the psychological distress and activity level subscales of the RSCL before testing structural regression paths [30].

**Fig 2:**
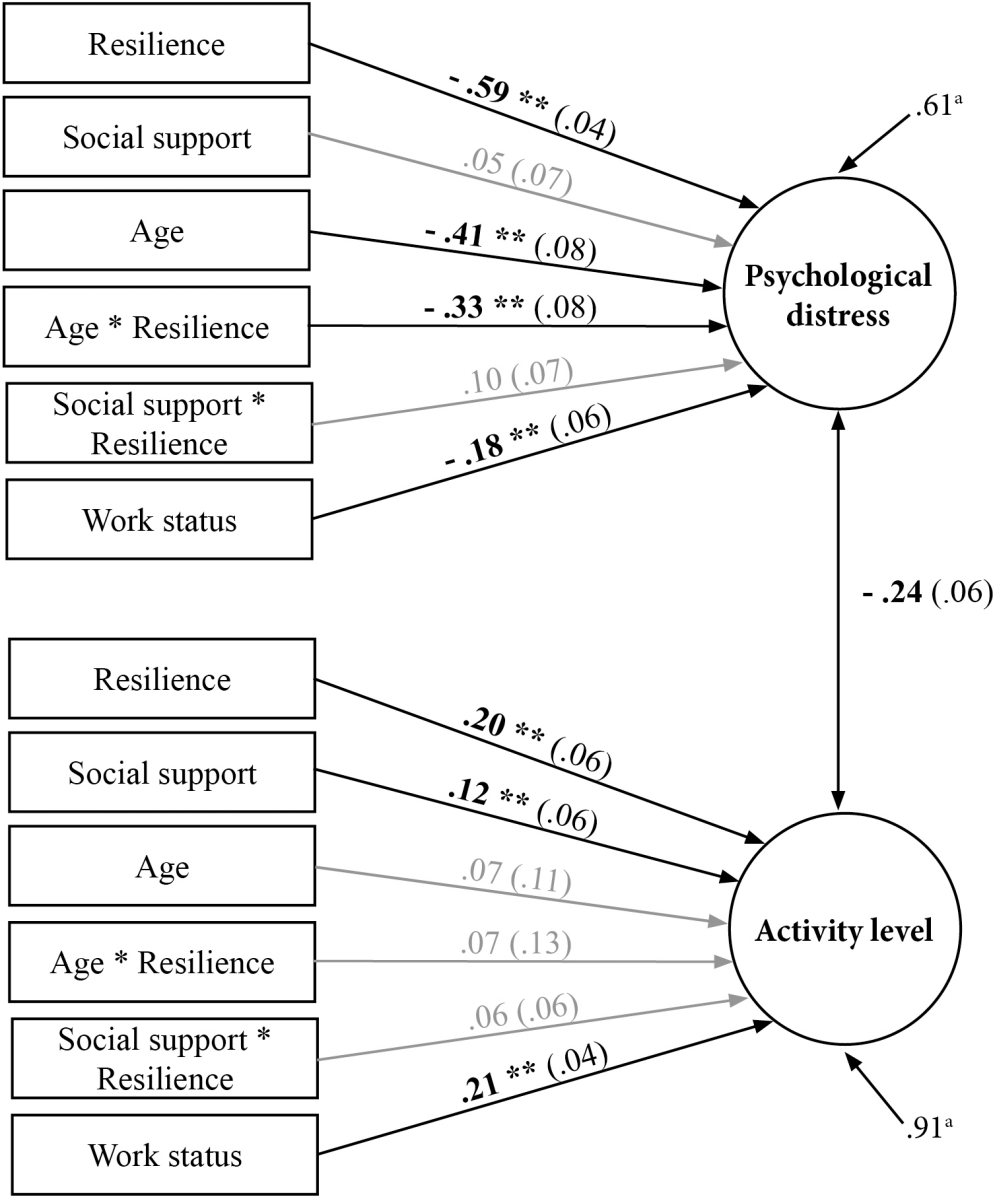
Statistical diagram of the revised model. **Relationship between resilience (Connor-Davidson Resilience Scale) psychological distress and activity level (Rotterdam Symptom Checklist).** Regression paths for age and social support are also shown, controlling for work status in the analysis. *Note*. Resilience*Age, Resilience*Social support: the moderating effects of age and social support. Standardized coefficients and standard errors are shown. ^a^Residual error in the prediction of the latent variable. **p < 0.01

Analyses were conducted using MplusV7.11. We employed full information maximum likelihood (FIML), to account for missing data [31]. Maximum likelihood estimator with robust errors (MLR) was used to correct for the skewed distribution in social support. Statistically significant moderation paths (see Fig 2) were probed using PROCESS, an add-on for SPSS [32]. A p-value of <0.05 was considered statistically significant.

## Results

During the 2-week recruitment period approximately 1100 patients were treated at the tertiary center. In total, 343 (37%) had available relevant data and were included in this analysis. Patient characteristics are presented in Table 1. Briefly, patients ranged in age from 19–88 years (median = 60 yrs) and spanned a range of educational level. Cancer diagnoses were varied. Approximately two-thirds of patients had invasive/metastatic/recurrent tumors and approximately three-quarters of patients were receiving chemotherapy. The majority of patients were in early or mid-treatment stages.

**Table 1:** Descriptive data: Clinical and socio-demographic variables of the sample. (*N* = 343).

*N = 343*	*%*
**Age: mean, SD**	58 ± 14.4 yrs (99%)
**Gender (male: female)**	41%: 59%
**Marital status**	
Married/living with a partner	66%
Single	13%
Divorced	12%
Widowed	9%
**Work status**	
Retired	54%
Not working/sick leave	29%
Working ≤ 15h/week	2%
Working 16h to 30h/week	3%
Working ≥ 30h/week	9%
**Living arrangement**	
No underage children in the home	86.3%
Underage children in the home	12.8%
**Education**	
Compulsory education	9%
Technical training	30%
Higher education	21%
University	23%
**Tumor site (per ICD-10 coding)**	
Lymphoid, hematopoietic and related tissue	23%
Breast	21.3%
Digestive organs	18.4%
Female genital organs	10.2%
Respiratory and intrathoracic organs	6.1%
Others	20.1%
**Tumor stage**	
Invasive, metastatic or recurrent tumor	66%
Non-invasive/ non-metastatic/ non-recurrent tumor	26%
**Treatment modality**	
Chemotherapy	74%
Radiotherapy	2%
Chemoradiation	24%
**Current chemotherapy cycle**	
Early treatment: 1–3 cycles	43%
Mid-treatment: 4–6 cycles	28%
Later treatment: ≥7 cycles	14%

Bivariate correlations among the study variables (Table 2) revealed that neither tumor stage nor marital status were correlated to any of the study variables, and were thus excluded from the Structural Equation Modelling (SEM) models. Gender, income, and work status were related to outcomes and other variables, so they were included in our initial model (S1 Table) to control for their influence. However, in our subsequent model (revised model) we removed covariate variables (gender and income) which were not statistically significant (S2 Table).

**Table 2:** Mean, standard deviation, and correlation coefficients of study variables (*N* = 343).

Variable	1	2	3	4	5	6	7	8	9	10
1.Psychological distress	1	-.28**	-.51*	.12*	-.10	.07	.08	-.03	-.04	-.10
2. Activity level		1	.22**	.03	-.20**	.02	-.16**	.12*	.05	.33**
3. Resilience			1	-.23**	-.02	.05	-.08	.13*	-.06	.07
4. Total Social support				1	.09	.08	-.12*	-.15*	.24**	-.04
5. Age					1	.02	-.12*	-.01	-.14*	-.46**
6. Tumor stage^a^						1	.02	.04	-.04	.03
7. Gender^b^							1	-.12*	.09	-.01
8. Income								1	-.36**	.28**
9. Marital status^c^									1	-.06
10. Work status										1
*Mean*	12.89	25.20	29.34	1.44	58.05					
*Median*	12.00	27.00	30.00	1.25	60			€1,500 – €2,600		1
*SD*	4.45	5.68	7.03	0.59	14.44					

^a^Tumor stage was coded as 1 = noninvasive, 2 = invasive tumor

^b^Gender was coded as 1 = male, 2 = female

^c^Marital status was coded as 1 = married/living with a partner, 2 = single/divorced/widowed; income (monthly) was coded as 1 = < €900, 2 = €900–1,500; 3 = €1,501–2,600, 4 = €2,601–4,000, 5 = > €4,000; Work status was coded as 1 = retired, 2 = unemployed/sick leave, 3 = < 15 hours per week, 4 = < 30 hours per week, 5 = > 30 hours per week

* p < 0.05

** p < 0.01.

### Resilience is negatively associated with psychological distress

The exploratory factor analysis (i.e. our measurement model) of the Rotterdam Symptom Checklist items related to psychological distress and activity level demonstrated acceptable fit statistics (χ^2^(79) = 192.420, *p* = .000, comparative fit index (CFI) =. 967, the Tucker-Lewis index (TLI) = .950, root mean square error of approximation (RMSEA) = .06, 90% CI [.049.071]). The two factors derived showed a moderate correlation (r = -.275), indicating some overlap between psychological distress and activity level. We then tested our initial SEM model (S1 Table) including resilience, age and social support as well as the interactions of resilience with both age and social support (controlling for the effects of gender, income, and work status). This model enabled us to predict both psychological distress and activity level. Neither gender nor income regression coefficients were statistically significant, so these were excluded in the revised model. The revised model (Fig 1, S2 Table), demonstrated a good model fit (χ^2^(163) = 313.76, *p* = .000, CFI = .942, TLI = .923, RMSEA = .053, 90% CI [.044.062]). While social support moderator paths (Psychological distress: β = .10, *p* = .12; Activity level: β = .06, *p* = .35) were not significant, we did not have substantive reasons to exclude them from our model, which, in addition, would have resulted in a only marginally better model fit. Several significant findings emerged from the simple effects. First, the negative association between resilience and psychological distress (β = -.59, *p* < .01, 95% CI: -.67, -.50) and the positive association of resilience with activity level (β = .20, *p* < .01, 95% CI: .08, .31). Second, the positive association of social support with activity level (β = .12, p < .05, 95% CI: .00, .23). Third, the negative association between work status with psychological distress (β = -.18, p < .01, 95% CI: -.28, -.06) and the positive association of work status with activity level (β = .21, p < .01, 95% CI: .13, .27). The positive association of social support with psychological distress (β = .05, 95% CI: -.09, .16) was not statistically significant. Thus, resilience had the strongest association with psychological distress and the second strongest with activity level, as work status had a slightly greater contribution.

### The relationship between resilience and psychological distress is moderated by age

**Fig 3:**
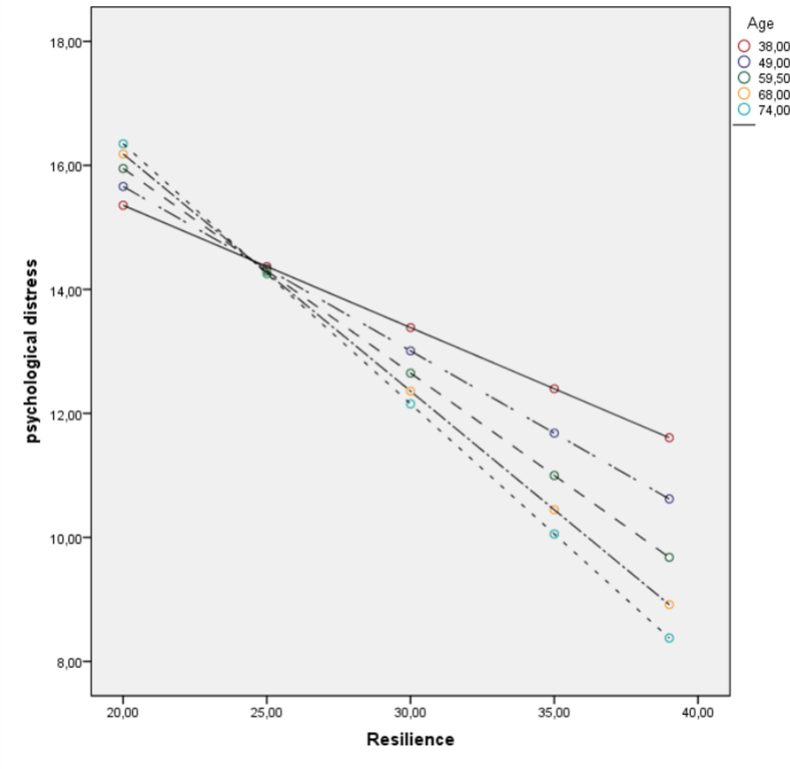
Moderating effect of age on the relationship between resilience and psychological distress. The moderator effects by age showing that compared to younger patients, older patients with equivalent levels of resilience (measured by CD-RISC 10) have lower levels of psychological distress (measured by RSCL). *Note*. The values of age represent the 10^th^, 25^th^, 50^th^, 75^th^ and 90^th^ percentiles in the sample distribution of age.

Age had a statistically significant negative association with psychological distress (β = -.41, *p* < .01, 95% CI: -.56, -.25), but not activity level (β = .07, 95% CI: -.14, .28). We tested the moderating effect of age on the link between resilience and the two main outcomes (psychological distress and activity level). Concerning psychological distress, the moderation path was statistically significant (β = -.33, p < .01, 95% CI: -.49, -.16) (Fig 3), whereas for activity level it was not (β = .07, 95% CI: -.18, .32).

## Discussion

In this cross-sectional study we found resilience to be strongly associated with lower levels of psychological distress. To a lesser degree, we also showed resilience is associated with higher activity levels (i.e. functional status). These findings are in line with previous reports on the relationship between resilience and favorable mental health outcomes in patients with various cancer diagnoses and in diverse cultural contexts. A French study examined breast cancer survivors and women undergoing mammography with no prior cancer diagnoses. Dividing the sample into groups with high and low levels of resilience it was found that while groups did not differ in terms of mood disorder, those study participants with high resilience scores had significantly less anxiety and depressive comorbidity (5-fold lower) and were less likely to have an anxiety disorder (3-fold lower risk) [11]. Similar findings, that is a significant negative association of resilience with both depression and anxiety (psychological distress), were reiterated in a sample of hospitalized cancer patients undergoing treatment in South Korea [13], in samples of German [12] and Turkish survivors of stem cell transplantation (most of which were cancer survivors) [7] and in a sample of Chinese patients undergoing treatment for digestive cancer [8]. Given that conceptualizations of resilience may be significantly shaped by the cultural context of the individual [33], these corresponding findings in European and Asian countries are remarkable for indicating that central principles of the concept of resilience have, at least to some degree, relevance across cultures.

Recently resilience has been linked to physical activity in a large population-based study. The Health and Retirement Study showed that among nearly 11’000 Americans between the ages of 51 and 98 years, resilience protects against limitations in activities of daily living and significantly modifies the relationship between the onset of a new chronic condition and subsequent disability [15]. Among patients from the United States of America, a study of 83 adults with Parkinson disease showed that higher resilience correlates with less disability [16]. In survivors of hematopoietic cell transplantation (mostly cancer survivors) from the United States resilience was shown to buffer against permanent disability [17]. A similar link with better physical functioning was found in a sample of German survivors of stem cell transplantation [12], as well as in long-term cancer survivors from the United States [18].

This protective relationship between resilience and activity levels/disability found in non-oncologic populations and among cancer survivors is not clearly corroborated by our findings. However, some have posited that a healthy level of physical functioning can be defined as a positive outcome of resilience [34]. This may be attributable in part to the fact that two-thirds of participants in the present study had been diagnosed with an invasive, metastatic or recurrent cancer and they were receiving active cancer treatment. As such, these patients were exposed to physiological, as well as psychosocial stressors. Notably, the instrument we used to measure resilience (CD-RISC 10) focuses almost exclusively on resilience in the face of stress and psychosocial adversity [35]. Importantly, physical impairment accompanying cancer treatment (e.g. surgical wounds, peripheral neuropathy) may not be adequately compensated by resilience, thus weakening the statistical association between these two factors.

Social support is commonly cited as having a buffering or protective effect on distress and psychosocial adjustment [36, 37]. In our sample, ratings of perceived social support approached the highest attainable score (median = 57, mean 55 ± 6.8 of a possible 60), indicating that patients rated their social support as highly adequate. Yet, social support was only significantly associated with better activity level, but not with psychological distress. In addition, social support moderator paths did not exhibit significant associations with neither psychological distress nor activity level. Initially we assumed that these data may challenge the assumption of a linear relationship between perceived social support and patient outcomes such as physical functioning or distress (i.e. an increase in social support is associated with a proportional decrease of distress). As it has been reported in a study of patients with breast cancer, it is plausible that after a critical threshold of social support has been reached, further increases may only result in incremental benefits for the patient. [38]. However, this assumption was not supported by our data, as the quadratic effect of social support on our outcome measures (i.e. linear increases, followed by a plateau and then decreasing effects) was not significant (S3 Table). Thus, we may neither confirm the protective effect of social support on psychological distress nor moderation effects of social support in our sample.

Of note, work status, a variable we did not include in our hypothesis and conceptual model, was significantly associated with less psychological distress and better activity level. That is, being employed (i.e. still working or being on sick leave) while receiving treatment was associated with favorable patient outcomes in our sample. This is in line with research in cancer survivors, which identified high levels of unmet needs for support from occupational health personnel, but also high levels of support from coworkers as factors that contributed to (or decreased, respectively) the ability of cancer survivors to return to the workplace after their treatment [39, 40]. Accordingly, being employed while receiving treatment may have provided the patients in our sample with an additional source of support, rather than being an additional burden or source of concern. However, additional research is required to establish causality between these factors.

Interestingly, older age was strongly associated with less psychological distress and only marginally associated with lower activity level. This is in line with research reporting that older cancer patients experience less distress and are better adapted than younger patients (e.g. by using positive reappraisal) [19]. Prior studies have reported increased resilience scores with age [19, 41], yet we found no significant differences. Rather, age was found to be a significant and strong moderator of resilience and psychological distress. That is, older patients tended to experience less psychological distress than younger patients who had comparable levels of resilience. Importantly, this highlights that although resilience is generally associated with less psychological distress, age is a strong moderator of this association. Resilience has been viewed as a capacity that is developed over time in response to stressors and hardships of life [6]. From this perspective, older patients may have developed a broader spectrum of skills and resources during their lifetime or they may use them more efficiently to ward off psychological distress compared to younger patients. Thus, younger cancer patients may be in particular need of interventions to facilitate resilience and decrease psychological distress during cancer treatment. This effectively extends the goal of cancer treatment beyond mere survival.

## Limitations

We acknowledge several limitations of this study. First and foremost the cross-sectional nature of this study did not allow us to establish causality between the variables under investigation. Additional longitudinal research is required in order to prospectively identify predictors of psychological distress and activity levels. Second, we were not able to replicate the factor structure of the RSCL concerning the physical symptom distress subscale (i.e. symptoms did not load on any factors or cross-loaded on several factors), which was not the focus of our analysis. To incorporate this subscale in future research, the factor structure of the RSCL needs to be examined further. Third, being a secondary analysis of a study conducted for quality assurance, which aimed to assess a broad range of symptoms while minimizing response burden, we report on findings obtained with the RSCL. A more detailed and comprehensive assessment instrument might have been favorable for this secondary analysis, especially concerning psychological distress.

## Conclusion

The findings from this cross-sectional study deepen our understanding of patient-related factors influencing symptom management and supportive care interventions. In particular, these data help elucidate the relationships between resilience, psychological distress, and activity among patients undergoing cancer treatment. Assessing resilience and using these data to tailor interventions to address specific factors facilitating resilience may be a promising way to improve the effectiveness of symptom management interventions. Further work, including longitudinal observational studies and/or interventional clinical trials, is needed to define causality between resilience, psychological distress, and activity as well as to identify predictors for positive outcomes.

## Supporting Information

S1 Table Initial Model. **The effects of variables on psychological distress and activity level with age and social support as moderators.***Note*. B = standardized coefficient, CI = confidence interval, SE = standard error, LL = lower limit, UL = upper limit, df = degree of freedom, CFI = comparative fit index, TLI = the Tucker-Lewis index, RMSEA = root mean square error of approximation. Statistically significant (p < .05) coefficients are in bold.
(DOC)

S2 Table Revised Model. **The effects of variables on psychological distress and activity level with age and social support as moderators.***Note*. B = standardized coefficient, CI = confidence interval, SE = standard error, LL = lower limit, UL = upper limit, df = degree of freedom, CFI = comparative fit index, TLI = the Tucker-Lewis index, RMSEA = root mean square error of approximation. Statistically significant (p < .05) coefficients are in bold.
(DOC)

S3 Table Model showing the non-linear direct effect of social support and other variables on psychological distress and activity level with age and social support as moderators. *Note*. B = standardized coefficient, CI = confidence interval, SE = standard error, LL = lower limit, UL = upper limit, df = degree of freedom, CFI = comparative fit index, TLI = the Tucker-Lewis index, RMSEA = root mean square error of approximation. Statistically significant (p < .05) coefficients are in bold.
(DOC)
