# Supplementary material for: Relationship between Resilience, Psychological Distress and Physical Activity in Cancer Patients: A Cross-Sectional Observation Study
Source: PLoS One. 2016 Apr 28;11(4):e0154496. doi: 10.1371/journal.pone.0154496 (PMC4849643; doi:10.1371/journal.pone.0154496)
Supplement: S1 Table — The effects of variables on psychological distress and activity level with age and social support as moderators.Note. B = standardized coefficient, CI = confidence interval, SE = standard error, LL = lower limit, UL = upper limit, df = degree of freedom, CFI = comparative fit index, TLI = the Tucker-Lewis index, RMSEA = root mean square error of approximation. Statistically significant (p < .05) coefficients are in bold. (DOC) [file pone.0154496.s001.doc]

**S1 Table. Initial Model. The effects of variables on psychological distress and activity level with age and social support as moderators.**

| Structural model | | **B** | | **95% CI** | | **SE** | ***P* value** |
| --- | --- | --- | --- | --- | --- | --- | --- |
|  |  |  |  | ***LL*** | ***UL*** |  |  |
| **Psychological Distress** | |  | |  |  |  |  |
| Resilience | | **-0.63** | | **-.71,** | **-.54** | **0.04** | **0.00** |
| Social support | | 0.04 | | -.80 | -.16 | 0.06 | 0.50 |
| Age | | **-0.41** | | **-.56** | **-.25** | **0.08** | **0.00** |
| Gender | | 0.05 | | -.06 | .15 | 0.06 | 0.41 |
| Income | | 0.09 | | -.01 | .20 | 0.05 | 0.32 |
| Work status | | **-0.20** | | **-.32** | **-.07** | **0.06** | **0.00** |
| Age*Resilience | | **-0.31** | | **-.48** | **-.12** | **0.09** | **0.00** |
| Social support*Resilience | | 0.09 | | -.05 | .22 | 0.07 | 0.22 |
| **Activity level** | |  | |  |  |  |  |
| Resilience | | **0.17** | | **.05** | **.28** | **0.06** | **0.01** |
| Social support | | **0.13** | | **.01** | **.24** | **0.06** | **0.03** |
| Age | | 0.01 | | -.19 | .20 | 0.10 | 0.94 |
| Gender | | -0.06 | | -.19 | .06 | 0.07 | 0.34 |
| Income | | 0.03 | | -.09 | .15 | 0.06 | 0.62 |
| Work status | | **0.18** | | **.09** | **.26** | **0.04** | **0.00** |
| Age*Resilience | | 0.07 | | -.14 | .28 | 0.11 | 0.53 |
| Social support*Resilience | | 0.06 | -.07 | | .19 | 0.07 | 0.36 |
| **Goodness of fit statistics** | **χ^2^ *(df*)** | | ***p*** | | **CFI** | **TLI** | **RMSEA [CI]** |
|  | 321.521 (*191*) | | .000 | | .945 | .929 | .049  [.040, .059] |

*Note*. B = standardized coefficient, CI = confidence interval, SE= standard error, LL= lower limit, UL= upper limit, df =degree of freedom, CFI= comparative fit index, TLI = the Tucker-Lewis index, RMSEA = root mean square error of approximation. Statistically significant (*p* <.05) coefficients are in bold.
