# Supplementary material for: Relationship between Resilience, Psychological Distress and Physical Activity in Cancer Patients: A Cross-Sectional Observation Study
Source: PLoS One. 2016 Apr 28;11(4):e0154496. doi: 10.1371/journal.pone.0154496 (PMC4849643; doi:10.1371/journal.pone.0154496)
Supplement: S2 Table — The effects of variables on psychological distress and activity level with age and social support as moderators.Note. B = standardized coefficient, CI = confidence interval, SE = standard error, LL = lower limit, UL = upper limit, df = degree of freedom, CFI = comparative fit index, TLI = the Tucker-Lewis index, RMSEA = root mean square error of approximation. Statistically significant (p < .05) coefficients are in bold. (DOC) [file pone.0154496.s002.doc]

**S2 Table. Revised Model. The effects of variables on psychological distress and activity level with age and social support as moderators.**

| Structural model | | **B** | | **95% CI** | | **SE** | ***P* value** |
| --- | --- | --- | --- | --- | --- | --- | --- |
|  |  |  |  | ***LL*** | ***UL*** |  |  |
| **Psychological Distress** | |  | |  |  |  |  |
| Resilience | | **-0.59** | | **-.67** | **-.50** | **0.04** | **0.00** |
| Social support | | 0.05 | | -.09 | .16 | 0.07 | 0.61 |
| Age | | **-0.41** | | **-.56** | **-.25** | **0.08** | **0.00** |
| Work status | | **-0.18** | | **-.28** | **-.06** | **0.06** | **0.00** |
| Age*Resilience | | **-0.33** | | **-.49** | **-0.16** | **0.08** | **0.00** |
| Social support*Resilience | | 0.10 | | -.02 | .23 | 0.07 | .0.12 |
| **Activity level** | |  | |  |  |  |  |
| Resilience | | **0.20** | | **.08** | **.31** | **0.06** | **0.00** |
| Social support | | **0.12** | | **.00** | **.23** | **0.06** | **0.03** |
| Age | | 0.07 | | -.14 | .28 | 0.11 | 0.52 |
| Work status | | **0.21** | | **.13** | **.27** | **0.04** | **0.00** |
| Age*Resilience | | 0.07 | | -.18 | .32 | 0.13 | 0.60 |
| Social support*Resilience | | 0.06 | -.06 | | .18 | 0.06 | 0.35 |
| **Goodness of fit statistics** | **χ^2^ *(df*)** | | ***p*** | | **CFI** | **TLI** | **RMSEA [CI]** |
|  | 313.761 (*163*) | | 0.00 | | 0.942 | 0.923 | 0.053 [.044, .062] |

*Note*. B = standardized coefficient, CI = confidence interval, SE= standard error, LL= lower limit, UL= upper limit, df =degree of freedom, CFI= comparative fit index, TLI = the Tucker-Lewis index, RMSEA = root mean square error of approximation. Statistically significant (*p* <.05) coefficients are in bold.
