# Supplementary material for: Relationship between Resilience, Psychological Distress and Physical Activity in Cancer Patients: A Cross-Sectional Observation Study
Source: PLoS One. 2016 Apr 28;11(4):e0154496. doi: 10.1371/journal.pone.0154496 (PMC4849643; doi:10.1371/journal.pone.0154496)
Supplement: S3 Table — Note. B = standardized coefficient, CI = confidence interval, SE = standard error, LL = lower limit, UL = upper limit, df = degree of freedom, CFI = comparative fit index, TLI = the Tucker-Lewis index, RMSEA = root mean square error of approximation. Statistically significant (p < .05) coefficients are in bold. (DOC) [file pone.0154496.s003.doc]

**S3 Table.** **Model showing the non-linear direct effect of social support and other variables on psychological distress and activity level with age and social support as moderators.**

| Structural model | | **B** | | **95% CI** | | **SE** | ***P* value** |
| --- | --- | --- | --- | --- | --- | --- | --- |
|  |  |  |  | ***LL*** | ***UL*** |  |  |
| **Psychological Distress** | |  | |  |  |  |  |
| Resilience | | **-0.59** | | **-.67** | **-.50** | **0.04** | **0.00** |
| Social support | | 0.00 | | -.17 | .17 | 0.09 | 0.96 |
| Social support (quadratic) | | 0.06 | | -.11 | .23 | .09 | 0.51 |
| Age | | **-0.41** | | **-.56** | **-.25** | **0.08** | **0.00** |
| Work status | | **-0.17** | | **-.30** | **-.03** | **0.07** | **0.00** |
| Age*Resilience | | **-0.33** | | **-.49** | **-0.16** | **0.08** | **0.00** |
| Social support*Resilience | | 0.12 | | -.01 | .25 | 0.07 | .0.08 |
| **Activity level** | |  | |  |  |  |  |
| Resilience | | **0.20** | | **.08** | **.31** | **0.06** | **0.00** |
| Social support | | **0.19** | | **.01** | **.36** | **0.09** | **0.03** |
| Social support (quadratic) | | -0.11 | | -.28 | .06 | 0.09 | 0.26 |
| Age | | 0.07 | | -.14 | .28 | 0.11 | 0.52 |
| Work status | | **0.21** | | **.13** | **.27** | **0.04** | **0.00** |
| Age*Resilience | | 0.08 | | -.17 | .33 | 0.13 | 0.57 |
| Social support*Resilience | | 0.03 | -.08 | | .14 | 0.06 | 0.58 |
| **Goodness of fit statistics** | **χ^2^ *(df*)** | | ***p*** | | **CFI** | **TLI** | **RMSEA [CI]** |
|  | 335.395 (*177*) | | 0.00 | | 0.940 | 0.921 | 0.052 [.044, .061] |

*Note*. B = standardized coefficient, CI = confidence interval, SE= standard error, LL= lower limit, UL= upper limit, df =degree of freedom, CFI= comparative fit index, TLI = the Tucker-Lewis index, RMSEA = root mean square error of approximation. Statistically significant (*p* <.05) coefficients are in bold.
